# Venovenous Extracorporeal Life Support in Single-Ventricle Patients with Acute Respiratory Distress Syndrome

**DOI:** 10.3389/fped.2016.00066

**Published:** 2016-06-28

**Authors:** Alison B. Nair, Peter Oishi

**Affiliations:** ^1^Department of Pediatrics, University of California San Francisco, San Francisco, CA, USA; ^2^Cardiovascular Research Institute, University of California San Francisco, San Francisco, CA, USA

**Keywords:** venovenous extracorporeal life support, extracorporeal membrane oxygenation, single ventricle, congenital heart disease, acute respiratory distress syndrome, cannulation, thrombosis, anticoagulation

## Abstract

There is new and growing experience with venovenous extracorporeal life support (VV ECLS) for neonatal and pediatric patients with single-ventricle physiology and acute respiratory distress syndrome (ARDS). Outcomes in this population have been defined but could be improved; survival rates in single-ventricle patients on VV ECLS for respiratory failure are slightly higher than those in single-ventricle patients on venoarterial ECLS for cardiac failure (48 vs. 32–43%), but are lower than in patients with biventricular anatomy (58–74%). To that end, special consideration is necessary for patients with single-ventricle physiology who require VV ECLS for ARDS. Specifically, ARDS disrupts the balance between pulmonary and systemic blood flow through dynamic alterations in cardiopulmonary mechanics. This complexity impacts how to run the VV ECLS circuit and the transition back to conventional support. Furthermore, these patients have a complicated coagulation profile. Both venous and arterial thrombi carry marked risk in single-ventricle patients due to the vulnerability of the pulmonary, coronary, and cerebral circulations. Finally, single-ventricle palliation requires the preservation of low resistance across the pulmonary circulation, unobstructed venous return, and optimal cardiac performance including valve function. As such, the proper timing as well as the particular conduct of ECLS might differ between this population and patients without single-ventricle physiology. The goal of this review is to summarize the current state of knowledge of VV ECLS in the single-ventricle population in the context of these special considerations.

## Introduction

Extracorporeal life support (ECLS) has been used in patients with severe cardiopulmonary failure for 40 years (1, 2). In the 1970s, pioneering clinicians adapted ECLS from cardiopulmonary bypass used in the operating room as a treatment for respiratory failure that was unresponsive to conventional therapy in the intensive care unit (3). Contemporaneous studies showed a survival benefit from ECLS in neonatal but not adult populations with severe respiratory failure (4, 5). In neonatal patients, ECLS interrupted the pathophysiologic cycle of hypoxia, acidosis, and pulmonary hypertension (2). However, with improvements in patient selection and technology, along with the more recent CESAR trial that showed a survival benefit in adult patients randomized to receive care in ECLS centers, ECLS has now become an important therapeutic option for adults with acute respiratory distress syndrome (ARDS) as well (2, 6). Likewise, based on experience drawn from these two populations and a growing body of research, ECLS for pediatric ARDS has also been demonstrated to be a viable treatment alternative to conventional therapy (7, 8).

Extracorporeal life support for cardiac failure has simultaneously evolved in each of these patient populations. In all ages, survival is less than in patients on ECLS for respiratory failure, but has improved over time (9).^1^

Regardless of the indication, whether cardiac, respiratory, or cardiorespiratory failure, ECLS was conducted through venous and arterial cannulation [venoarterial (VA) ECLS] when it was first introduced into clinical practice. Progressively, experience increased with venovenous (VV) cannulation for patients with respiratory failure but preserved cardiac function. Initially, this strategy required two separate cannula be placed in two large veins (e.g., bilateral femoral venous cannulation). Today, most VV ECLS can be performed using single site cannulation with a dual-lumen catheter.

Single-ventricle patients with ARDS supported with VV ECLS exist at an interesting intersection of these experiences. They have significant cardiac disease, but require ECLS for a primary respiratory indication. The population spans the neonatal and pediatric age groups, and increasingly will include adults. Cannulation strategies must account for an anatomic spectrum between patients and changes that will occur within individuals as they progress through surgical palliation of their heart disease. This review explores the prevailing physiologic considerations relevant to this challenging patient population in the context of the published literature.

## ECLS in Respiratory Failure

The use of ECLS for respiratory failure in the pediatric population remained relatively stable until the Influenza A H1N1 outbreak in 2009 after which its use has increased each year (8–11). In neonates, ECLS peaked in 1992 and then decreased in large part related to the introduction of inhaled nitric oxide to clinical practice (8)^1^. In both neonatal and pediatric patients, survival has not changed significantly in recent years (74% neonatal survival, 58% pediatric survival)^1^. However, the trend is toward placing more complex and sicker patients on ECLS. For example, pediatric patients placed on ECLS with a comorbid condition increased from 19% in 1993 up to 47% in 2007 with a 20% increase in survival of these patients (11). Factors associated with mortality in patients with respiratory failure on ECLS include diagnosis (pertussis, ARDS related to sepsis, fungal pneumonia, congenital diaphragmatic hernia), comorbid conditions (liver failure, cancer, renal failure, cardiac arrest), duration of ventilation prior to initiation of ECLS, and pre-ECLS pH (10, 11). Some studies but not all have found VV ECLS to have a lower mortality that VA ECLS in respiratory failure patients (8, 11, 12).

## ECLS in the Single-Ventricle Population

Extracorporeal life support was first used in patients with single-ventricle physiology who experienced difficulty separating from cardiopulmonary bypass in the operating room. This was described in a 1996 outcome analysis of a single center’s experience with patients undergoing surgery for congenital heart disease placed on VA ECLS. Initial survival estimates were between 17 and 25% depending on the stage of palliation (13). Since that time, the description of VA ECLS in single-ventricle patients has evolved and become further refined. Numerous outcome studies have reported a wide range of survival depending on the year of study, institution, underlying anatomy, and stage of palliation (14–26). The most recent estimates suggest a survival of 32–43% with later stages of palliation having higher mortality (27, 28).

## VA VS. VV ECLS

Historically, all single-ventricle patients in need of more than conventional therapy were placed on VA ECLS regardless of indication. As technology and expertise grew in the area of VV ECLS for ARDS, it became a consideration for the unique single-ventricle population with respiratory failure and adequate cardiac function. There are several potential benefits to VV compared to VA ECLS in this population, but also challenges. First, in VV ECLS, fully oxygenated blood from the circuit augments the oxygen content of blood delivered to the lung, which may counteract the increased pulmonary vascular resistance (PVR) that can occur in ARDS (29, 30). In single-ventricle patients, potential differences in pulmonary oxygen content between VA and VV ECLS depend on the stage of palliation and cannula position. Clinicians must consider these potential differences since second and third stage palliation results in passive pulmonary blood flow, and preserving a low PVR may be particularly important. Second, because ventricular ejection provides all systemic blood flow in VV ECLS, pulsatile flow is preserved. Non-pulsatile flow has been shown to increase catecholamine release, which may impair flow through the microcirculation, increase myocardial work through increased systemic afterload, and decrease end-organ perfusion (29, 31). Third, VV ECLS might decrease the risk of stroke if blood from the circuit can be returned exclusively to the pulmonary circulation (29, 30). However, in single-ventricle patients, this situation is uncommon, occurring only in Glenn patients if the inflow cannula (for multisite VV ECLS) is placed in the SVC, in Glenn patients if a dual-lumen single cannula is placed in the SVC, and in patients with an unfenestrated Fontan. Thus in general, VV ECLS does not afford the usual advantage over VA ECLS of decreased stroke risk in single-ventricle physiology. Finally, VV ECLS flow rates should not directly impact the ratio of pulmonary to systemic blood flow (Qp:Qs). Thus, like all VV ECLS, pump flow can be titrated based on the percentage of cardiac output necessary to provide adequate oxygenation and ventilation for any given amount of recirculation. Conversely, in VA ECLS in order to maintain adequate systemic oxygen delivery, flow must increase in a manner proportional to the Qp:Qs. Indeed, depending on the native ventricular function, anatomy, cannula size, and cannula position, VA ECLS could result in a marked increase in pulmonary blood flow that could aggravate the underlying lung injury. Likewise, higher ECLS flow rates could result in more inflammation, hemolysis, and autoantibody formation (29). Several of these points will be discussed in further detail below.

## VV ECLS in Single-Ventricle Patients

The limited experience of VV ECLS in the single-ventricle population is summarized in Table 1 (18, 29, 30, 32, 33). Booth and colleagues (18) were the first to describe the use of VV ECLS in single-ventricle patients in 2004. In a retrospective report, they described a cohort of 20 single-ventricle patients with cavopulmonary connections supported on ECLS. Their cohort included two patients placed on VV ECLS for respiratory indications, one following bidirectional Glenn surgery who did not survive and one following Fontan surgery who required conversion to VA ECLS due to the development of sepsis and hemodynamic instability (18).

**Table 1:** **Summary of VV ECLS in single-ventricle patients reported in the literature**.

Study	Year(s)	*N* with single-ventricle anatomy placed on VV ECLS	Single-ventricle patient diagnosis	Stage of palliation Percent (*n*)	Indication Percent (*n*)	Cannulation Percent (*n*)	Survival Percent (*n*)	Complications Percent (*n*)
Booth et al. (18)	1984–2002	1	DILV	BDG	Post-operative cyanosis	Right IJ and CA	No	Death

		1	Hypoplastic RV	Fontan	RSV pneumonia	Right FV	Yes	Developed sepsis, required conversion to VA ECLS
		In full cohort: *n* = 20 (2 VV and 18 VA ECLS SV patients)					In full cohort: 40% (8)	

Imamura et al. (30)	1997–2003	9	4 HLHS 2 PA/IVS 3 TA	In full cohort: 47% (8) Systemic to pulmonary shunt 18% (3) Other surgery 35% (6) No surgery	In full cohort: 41% (7) Acute viral pneumonia 59% (10) Acute severe hypoxia	In full cohort: 82% (14) RA/CA via right IJ 18% (3) RA/CA via transthoracic approach	In full cohort:88% (15)	In full cohort: 59% (10) required surgical intervention to wean from VV ECLS support
		In full cohort: *n* = 17 (9 SV and 8 biventricular patients on VV ECLS)						

Ryan et al. (32)	2010	1	HLHS	BDG	Post-operative cyanosis	CA via transthoracic approach	Yes	None

Jolley et al. (33)	1999–2012	4	ND	BDG	ND	ND	100% (4)	ND
		In full cohort: *n* = 103 (4 VV and 99 VA ECLS SV patients)					In full cohort: 41% (42)	

Aydin et al. (29)	1990–2012	89	22 HLHS 67 Other SV	14% (13) No surgery 61% (54) Shunt physiology 25% (22) Classic Glenn, BDG, Fontan	34% (30) Cardiac 66% (59) Respiratory 9% (8) Other	64% (57) IJ 27% (24) RA/CA 11% (10) FV	48% (43)	Most common: 25% (22) Surgical bleeding 42% (37) Renal injury 47% (42) Cardiac support with inotropes
					All sites included for patients with multisite cannulation			

*BDG, bidirectional Glenn; CA, common atrium; DILV, double inlet left ventricle; FV, femoral vein; HLHS, hypoplastic left-heart syndrome; IJ, internal jugular vein; ND, not described in the publication; PA/IVS, pulmonary atresia with intact ventricular septum; RA, right atrium; RSV, respiratory syncytial virus; RV, right ventricle; SV, single ventricle; TA, tricuspid atresia*.

Imamura and colleagues (30) reported a case series later that year of 17 patients with cyanotic heart disease placed on VV ECLS for either acute hypoxia or pneumonia. Nine of these patients had single-ventricle anatomy. The full cohort had a high survival rate with only two mortalities due to late sepsis after decannulation. Fifty-nine percent of these patients required a surgical procedure to wean off ECLS (30).

Six years later, Ryan and colleagues (32) described a patient following bidirectional Glenn surgery who survived VV ECLS. This patient was prenatally diagnosed with hypoplastic left-heart syndrome (mitral atresia and aortic atresia), a restrictive atrial septum, and aortic arch hypoplasia. He had an uncomplicated course through his bidirectional Glenn procedure after which he developed refractory hypoxemia despite normal ventricular function, low transpulmonary pressures, and low atrial pressures. He was supported for 7 days on VV ECLS with improvement in his arterial saturations and no significant change to his pulmonary pressures or cardiac function on subsequent cardiac catheterization (32).

Aydin and colleagues (29) provided the most detailed description to date of VV ECLS in single-ventricle patients. Using data reported to the Extracorporeal Life Support Organization (ELSO) from 1990 to 2012, they described 89 single-ventricle patients at various stages of surgical palliation placed on VV ECLS. Their cohort had a 48% survival to discharge with duration of intubation before initiation of ECLS, mean airway pressure and partial pressure of carbon dioxide prior to cannulation, and renal injury all associated with mortality (29). While this is the largest published report in this patient population and suggests that VV ECLS is a viable option in single-ventricle physiology, there are questions that could not be answered by these data. Specifically, 34% of patients in this cohort were placed on VV ECLS for “cardiac reasons”. Because this is a descriptive retrospective registry report, the authors could not provide more details about this group of patients. VV ECLS does not directly support cardiac function, and, thus, it is unknown whether there are clinically important distinctions between patients identified as needing support for cardiac vs. respiratory indications, or if these distinctions are due to vagaries in patient coding (29, 34, 35). There is also no information on the timing of ECLS in relation to surgery. Presumably there are significant differences between patients supported with ECLS in the immediate post-operative period and those placed on ECLS much longer after surgery (29, 35). Finally, there are no data on the rate of conversion from VV to VA ECLS in this cohort, an important outcome when considering an ECLS approach in these patients (29, 34).

## Special Considerations in Single-Ventricle ARDS Patients on VV ECLS

### Timing of Cannulation

Though the use of mechanical ventilation in ARDS is potentially lifesaving, the associated cyclic regional over-distention and alveolar collapse, along with toxicity from the high-inspired oxygen that is necessary to compensate for impaired gas exchange can all worsen the underlying lung injury. Though lung-protective ventilation can mitigate ventilator-associated lung injury and oxygen toxicity, these forces are still present and mortality in ARDS using conservative strategies is significant (6, 36). ECLS has the potential to greatly reduce if not eliminate these factors. The proper timing of ECLS remains a difficult clinical dilemma, which must balance consideration of the potential benefit of lung rest afforded by ECLS against its associated morbidities.

Multiple observational adult studies have found outcomes to be associated with duration of mechanical ventilation prior to initiation of ECLS in patients with ARDS (37–40). For example, Pranikoff and colleagues (37) found that survival in adults with ARDS was inversely associated with the number of pre-ECLS mechanical ventilation days and a 50% mortality at 5 days of mechanical ventilation. Beiderlinden and colleagues (38) found the average number of pre-ECLS mechanically ventilated days to be 5.3 in survivors as compared to 8.7 in non-survivors. The current adult ARDS recommendations suggest that patients mechanically ventilated for more than 7 days may be less likely to benefit from ECLS for respiratory failure (2, 36).^2^

Zabrocki and colleagues (11) evaluated this question in the pediatric ARDS population. They found that patients ventilated for ≤14 days had similar survivals between 56 and 61%, while those that were ventilated >14 days significantly dropped their survival to 38%. Of note, the group of patients ventilated between >7 and 14 days had lower but not significantly lower survival than those ventilated 0–7 days, so there may be important survival differences that were not able to be differentiated in this study. Indeed, Nance and colleagues (41) reported a statistically significant survival decrease of 2.9% for each pre-ECLS ventilator day (41). As such, the most current ELSO guidelines for pediatrics suggest that consideration of ECLS is best within the first 7 days of mechanical ventilation at high levels of support (2, 11).^3^ At this point, a clear consensus is lacking on the proper timing of VV ECLS in patients with respiratory failure.

In the single-ventricle VV ECLS population, Aydin and colleagues (29) showed that a shorter duration of intubation prior to initiation of VV ECLS was associated with mortality. Specifically, they found the median duration of intubation prior to ECLS in survivors to be 24 h as compared to 76 h in non-survivors (*p*-value = 0.004) with an odds ratio on multivariate analysis of mortality to be 1.01 (95% CI 1.003–1.016, *p*-value = 0.003) (29). Important physiology underlies the question of timing of cannulation in the single-ventricle patient. As mentioned, these patients rely on a low PVR and transpulmonary gradient at later stages of palliation. Pulmonary vascular dysfunction is known to occur in patients with ARDS (42). Furthermore, elevations in PVR and transpulmonary gradient have been shown to be independent predictors of mortality in ARDS, even in patients without heart disease (42). Not only is elevated PVR a common occurrence, but also PVR tends to drop in survivors and remains elevated in non-survivors (43). Mechanistically, endothelial injury resulting in inflammation, thrombosis, increased vascular tone due to hypoxic pulmonary vasoconstriction and an imbalance of vascular mediators, and pulmonary vascular remodeling with intimal fibrosis are all key in the elevation of PVR (44, 45). The consequences of these changes could be devastating for patients awaiting palliation with cavopulmonary connections as well as those already dependent on passive pulmonary blood flow. Thus, although clinical evidence is lacking, there is sound rationale for ECLS in patients with single-ventricle physiology and ARDS, perhaps even very early in the course of illness. Further research is required to conclusively demonstrate the proper timing of ECLS in these patients.

### Cannula Type and Location

The experience with cannula placement continues to evolve in VV ECLS. The most comprehensive review of cannula type in the overall VV ECLS pediatric population came from Zamora and colleagues (46). Using the ELSO database, they compared single dual-lumen venovenous cannulas (VVDL) and multisite venovenous (VVMS) cannulation. Over the 14-year cohort, they found similar overall utilization of VVDL and VVMS though the annual use of VVDL was increasing, reaching 71% of all cannulas in 2011. VVDL strategies were able to achieve higher flow rates overall. Survival was similar between the two groups and they found no difference in outcomes between wire-reinforced and non-wire-reinforced cannulas. Importantly, mechanical and cardiovascular complications were higher in patients with VVDL cannula (46). The higher incidence of cardiovascular complications is a potential concern in single-ventricle patients who tend to have more fragile hemodynamics.

Indeed, cannula type and location are a particular challenge in single-ventricle patients. In patients at the first stage of palliation with a Sano shunt, special care should be taken so that cannula placement does not mechanically interfere with flow through the shunt. In addition, for all patients at the first stage of palliation, it is critical to consider that unobstructed SVC flow is necessary for second-stage palliation with a Glenn (superior cavopulmonary connection) and unobstructed IVC flow is necessary for third-stage palliation with a Fontan (total cavopulmonary connection). As such, the ramifications of vascular injury or occlusive thrombus are far graver in these patients than in patients with biventricular physiology. Furthermore, for patients at these stages of palliation, it is important to ensure that the superior and inferior caval circulations have sufficient cerebral and lower body drainage and perfusion. Inability to maintain adequate drainage and perfusion has been attributed to worse outcomes in this population (18, 33).

According to Aydin and colleagues (29), in the single-ventricle VV ECLS population, VVDL was used in 70% of patients with the internal jugular vein being the most common cannulation site (64%). The most common cannula approaches based on anatomy were (1) VVDL placed in the right internal jugular vein for unrepaired single-ventricle patients or those with a central or Sano shunts, (2) VVMS in the right internal jugular and femoral vein for those with a classic or bidirectional Glenn shunt, and (3) VVMS in the right internal jugular and femoral vein in patients with a Fontan. Importantly, there was no association with mortality between cannula types and cannula sites (29). Cannulation sites in all VV ECLS single-ventricle studies are included in Table 1. Breakdown of cannula type and location according to Aydin and colleagues (29) is shown in Table 2.

**Table 2:** **Cannula type and location in VV ECLS single-ventricle patients (29)**.

		Survivors (*n* = 46)	Non-survivors (*n* = 43)	*p*-value
Cannula type	Venovenous double lumen (VVDL)	27 (59%)	30 (70%)	0.28
	Venovenous (VV) not otherwise specified	17 (37%)	10 (23%)	0.15
	Venovenous double lumen with additional single-lumen venous cannula (VVDL-V)	2 (4%)	3 (7%)	0.53

		Survivors (*n* = 46 patients, 51 cannulations)*	Non-survivors(*n* = 43 patients, 48 cannulations)*	***p*-value**

Cannulation location	Jugular vein	30 (59%)	27 (56%)	0.76
	Right atrium	12 (23%)	12 (25%)	0.82
	Femoral vein	5 (10%)	5 (11%)	0.87
	Other	4 (8%)	4 (8%)	1

*Percentage based on number of cannulations.

The proper cannulation strategy for single-ventricle patients at particular stages of palliation remains a critical unanswered question that requires more study with both short- and long-term outcome measures. In the absence of guiding data, careful case-specific consideration is required that accounts for the particular anatomy, ECLS flow requirements, and future surgical procedures in order to make the best determination of the risks and benefits of any given cannulation strategy. Furthermore, since patients with single-ventricle physiology have limited physiologic reserve and techniques used to measure cardiac function have limitations, clinicians must be prepared to transition to VA ECLS after embarking on a VV strategy as the initial approach if the clinical response is inadequate.

### Bleeding, Thrombosis, and Anticoagulation

In general, patients with single ventricles need special consideration in regard to coagulation. These patients are at risk for thrombosis perioperatively related to their surgery and post-surgical management. They are also at risk between stages of palliation due to interference with laminar flow caused by altered anatomy, potential cardiac dysfunction, and the introduction of shunts, sutures, and other thrombogenic artificial material that creates an environment suitable for thrombus formation (47). Procoagulant and anticoagulant abnormalities have been found in patients at all stages of palliation. Preoperatively, patients tend to have low levels of procoagulants and anticoagulants with different studies relating these deficiencies to oxygen saturation and ventricular dysfunction (48, 49). Interestingly, these patients do not seem to be predisposed to bleeding, suggesting that their factor and protein suppression maintains hemostatic balance (47). Post-operatively, both Glenn and Fontan patients transiently develop coagulopathy related to hemodynamic changes and liver dysfunction (50). Abnormal hemodynamics in the absence of a ventricular chamber dedicated to pumping venous return into the pulmonary vascular bed may predispose patients to subclinical hepatic dysfunction, leading to selective disturbances of protein synthesis.

While the clinical significance of these coagulation abnormalities is not directly known, the epidemiologic data suggest that single-ventricle patients have a predisposition for thrombosis. Manlhiot and colleagues (47) reported thrombotic risk after all three stages of palliation. The highest risk comes after the first stage of palliation with thrombotic complications occurring in 40% of patients. An important component of these thrombotic complications is Blalock–Taussig shunt thrombus formation, which has a reported incidence of 1–17%. Following the second stage of palliation, thrombotic complications occur in 28% of patients. The 5-year freedom of thrombotic complications was 79% in Fontan patients. Of note, thrombotic complications were associated with increased mortality after all stages (47).

Extracorporeal life support only adds to the complicated hemostatic picture in this patient population. Bleeding and thrombosis are common complications in ECLS with one or both seen on 86% of autopsies done after ECLS-related mortality (51). Based on the ELSO registry between 2005 and 2011, clinical bleeding complications occurred in 38% of patients, while thrombosis was noted in 31% of patients on ECLS. Furthermore, survival was decreased by 40% when a bleeding complication occurred and by 33% when a thrombotic complication occurred. Factors associated with bleeding and thrombosis included longer duration of ECLS and use of VA cannulation (52). Specifically, in pediatric cardiac surgery patients on ECLS, hemorrhagic complications occurred in 57% of patients and their mortality was higher than those without (53). ECLS is also known to be associated with increased odds of thrombus formation in this population (54).

Aydin and colleagues (29) retrospectively addressed this question in the VV ECLS single-ventricle population. They found thrombus related to the ECLS circuit in 18% of patients, surgical bleeding in 25% of patients, and hemolysis in 5% of patients. There was no difference in incidence between survivors and non-survivors (29). Interestingly, these initial data suggest a lower risk of bleeding and thrombotic complications than is seen in both the pediatric cardiac surgery patients on ECLS and in the broader ECLS population. More studies are required to better understand these differences.

In sum, single-ventricle patients have a physiology that supports a predisposition to thrombus formation, abnormal coagulation profiles, and a higher observed incidence of thrombus and bleeding. The risks of bleeding and thrombosis associated with ECLS contribute to the already complex coagulation considerations in the single-ventricle patient.

### VV ECLS Flows in Single-Ventricle Patients

Patients with aortopulmonary shunts require high flows on VA ECLS because of pulmonary runoff. Supporting higher flows can be achieved with large cannula or multiple cannulation sites, but may be technically difficult to achieve as these patients are usually infants and may have abnormal, stenotic, or thrombosed vasculature. Strategies to control pulmonary blood flow can include medical interventions, such as high positive end-expiratory pressure (PEEP), low-inspired oxygen, and permissive hypercapnia. Surgical interventions include restriction of the shunt with a clip or ligature (26). Full occlusion of the shunt to prevent pulmonary run off has been described and found to be associated with poor survival (15, 26). In addition, there is a potential risk for the arterial cannula to enter or occlude a shunt. The use of VV ECLS when cardiac function is adequate decreases the risk for over-circulation in turn obviating the need for these medical or surgical strategies to limit pulmonary blood flow and their potential complications. Furthermore, lower flows decrease patient exposure to blood products, lessen hemolysis risk, and reduce the activation of inflammation by decreasing blood contact with the ECLS circuit.

### Lung Rest Settings

Data are lacking across patient populations on ideal mechanical ventilator settings for patients on ECLS (55, 56). In fact, in some patient populations (e.g., patients awaiting lung transplantation) extubation while on ECLS is an emerging management strategy (55, 57). Although VV ECLS can replace lung function, this depends upon the efficiency of ECLS, which relates to the maximum achievable flow and the extent of recirculation. Therefore, at times some ventilator support might be required to augment ventilation and/or oxygenation (55). Beyond these considerations, important questions remain unanswered regarding ideal lung rest ventilator settings (58). For example, what is the ideal physiology for lung recovery? In general, based on published ECLS trials (that described but did not study ventilator settings) higher levels of PEEP, lower inspired oxygen, low tidal volumes, lower peak and plateau pressures, and lower rates appear to be common practice (54, 56, 59, 60). The single-ventricle population raises further questions. In patients at the first stage of palliation, it might be most appropriate to manage the ventilator in a manner that avoids increasing the Qp:Qs. In patients at the second and third stage of palliation (i.e., Glenn and Fontan), the impact of pulmonary venous return on cardiac output is an important factor in the ventilator management. Further study is needed to optimize this aspect of management.

### Weaning Off of ECLS

Similar to ideal lung rest settings, a standard process for weaning off of ECLS is lacking. Trials off of VV ECLS are far simpler than trials off of VA ECLS, since clinicians can simply stop delivering sweep gas through the oxygenator without disconnecting the patient from the ECLS circuit (58). Conversely, weaning from VA ECLS requires reduction in circuit flow and the introduction of a bypass bridge if a trial off all flow is desired. For patients with single-ventricle physiology, clinicians must carefully consider the respiratory and circulatory status of the lung in order to ensure that the benefit of ECLS has been fully leveraged. Readiness for separation from ECLS differs fundamentally between single-ventricle patients and most patients supported with ECLS for respiratory failure. Impaired cardiopulmonary interactions are poorly tolerated in these patients, requiring careful scrutiny of the adequacy of systemic oxygen delivery during the transition from lung rest settings to full ventilation. Given the challenges associated with cannulation in these patients, reinstituting ECLS after decannulation would be expected to be difficult or not possible. As such, longer weaning trials (off of sweep gas flow prior to decannulation) may be wise.

## Conclusion

Based on growing experience, VV ECLS to support the single-ventricle patient with ARDS is a viable option when conventional therapy fails or is associated with significant morbidity. Outcomes are within the scope of ECLS outcomes in other patient populations. Special thought is necessary given the unique single-ventricle physiology, including cannulation, hemorrhagic, thrombotic, circuit flow, and lung rest considerations. In addition, more study is necessary to further understand and enhance the management of these patients.

## Author Contributions

AN’s contributions include (1) substantial contributions to the conception or design of the work; or the acquisition, analysis, or interpretation of data for the work, (2) drafting the work or revising it critically for important intellectual content, (3) final approval of the version to be published, and (4) agreement to be accountable for all aspects of the work in ensuring that questions related to the accuracy or integrity of any part of the work are appropriately investigated and resolved. PO’s contributions include (1) substantial contributions to the conception or design of the work; or the acquisition, analysis, or interpretation of data for the work, (2) drafting the work or revising it critically for important intellectual content, (3) final approval of the version to be published, and (4) agreement to be accountable for all aspects of the work in ensuring that questions related to the accuracy or integrity of any part of the work are appropriately investigated and resolved.

## Conflict of Interest Statement

The authors declare that the research was conducted in the absence of any commercial or financial relationships that could be construed as a potential conflict of interest.

## Footnotes

^1^ECLS Registry Report. International Summary. Extracorporeal Life Support Organization. http://www.elso.org/Registry/Statistics.aspx. Accessed April 2016.

^2^ELSO Guidelines for Adult Respiratory Failure v1.3. Extracorporeal Life Support Organization. http://www.elso.org/resources/Guidelines.aspx. Accessed April 2016.

^3^ELSO Guidelines for Pediatric Respiratory Failure v1.3. Extracorporeal Life Support Organization. http://www.elso.org/resources/Guidelines.aspx. Accessed April 2016.
